# The Impact of System-Related Magnetic Resonance Imaging Geometric Distortion in Stereotactic Radiotherapy: A Case Report

**DOI:** 10.7759/cureus.27269

**Published:** 2022-07-26

**Authors:** Motoki Kumagai, Mariko Kawamura, Yutaka Kato, Kuniyasu Okudaira, Shinji Naganawa

**Affiliations:** 1 Department of Radiology, Nagoya University Graduate School of Medicine, Nagoya, JPN; 2 Department of Radiological Technology, Nagoya University Hospital, Nagoya, JPN

**Keywords:** distortion correction, radiotherapy treatment planning, brain metastases, distortion, magnetic resonance imaging, stereotactic radiotherapy

## Abstract

Magnetic resonance imaging (MRI) is now essential in stereotactic radiotherapy (SRT) planning for brain tumors because of its excellence in soft-tissue contrast and high spatial resolution. However, MRI distortion is sometimes difficult to recognize, and it may cause large misalignments in radiotherapy planning. In this case report, we will show how much difference in the dose distribution of SRT can be made by using MRI without distortion correction.

## Introduction

Stereotactic radiotherapy (SRT) for limited brain metastases is now an established treatment used in daily practice [1-4]. The accuracy of SRT has been improved [5], and irradiation is now possible with an accuracy of 1 mm or less.

However, quality assurance for tumor delineation is far from sufficient. In recent years, attempts have been made to achieve a more accurate delineation of tumors. One of these strategies is the use of magnetic resonance imaging (MRI), which is particularly useful for the delineation of brain tumors [6]; its use is invaluable in stereotactic irradiation of the brain.

Several validation reports have been published on MRI distortion [7-9], and caution should be exercised when using MRI in radiation treatment planning. This is especially true for 3T MRI, which is suitable for evaluating detailed structures of the brain. However, when the MRI is far from the central axis, there is a large amount of distortion that causes spatial misalignment [10].

During treatment planning for brain tumors, CT and MR images are often fused with reference to the bone structure to enable a more accurate delineation of target volumes and normal structures. However, if the bone and tumor are spatially distorted, the actual location of the tumor (the location of the tumor on the CT image) and the location where the tumor appears to be on the MRI will diverge without being recognized by the radiation oncologist or physicist who is planning the SRT.

The purpose of this case report is to remind physicians and physicists of the importance of distortion correction by demonstrating how difficult it is to recognize large misalignments when distortion is not corrected in diagnostic MRI images that are fused with CT for treatment planning.

## Case presentation

A 52-year-old woman with a metastatic brain tumor from choriocarcinoma was referred to our hospital to receive stereotactic radiotherapy for the brain metastases.

Treatment planning

Computed Tomography Simulation and Magnetic Resonance Images With Distortion Correction

A 1-mm thickness slice CT simulation scan (SOMATOM Confidence RT Pro, Siemens, Erlangen, Germany) was obtained with the patient in a supine position with a thermoplastic immobilizing mask (Uni-frame, CIVCO Radiotherapy, Coralville, Iowa). The voxel size of the CT data set was 1.17 X 1.17 X 1.00 mm. The MR images were obtained by a 3T scanner (MAGNETOM Skyra, Siemens) using a 32-channel array head coil. Images were obtained based on a 3D T1-weighted gradient echo sequence with gadolinium enhancement. The precise scan parameters used were: repetition time, 1570 ms; echo time, 2.29 ms; flip angle, 15°; field of view, 233 × 233 mm; matrix size, 256 × 256; slice thickness, 0.9 mm; slices per slab, 224; bandwidth, 200 Hz/pixel; parallel-imaging acceleration factor, 3; number of averages, 1; inversion time for non-selective inversion pulses, 800 ms. The acquisition time was 2 min 53 sec. Images were obtained without applying distortion correction (DC) and subsequently reconstructed using vendor-specific 3D-DC, whose accuracy we have previously reported [11].

Radiotherapy Planning

Brain metastasis was defined as gross tumor volume (GTV) coinciding with clinical target volume (CTV) based on the T1 contrast sequence of MRI on rigid co-registration with CT simulation. For actual treatment planning, we used MRI with DC. We added a 2-mm planning target volume (PTV) margin to the CTV and prescribed 14.4 Gy in three daily fractions, which was 36.7% of the maximum dose. The maximum dose was 39 Gy within the GTV using Precision version 3.3. The treatment was delivered using CyberKnife (Accuray Inc., Sunnyvale, CA, USA). Since there was a history of whole brain irradiation prior to SRT, the plan was made to increase the central dose while decreasing the limbic dose.

Comparison of the Treatment With and Without Magnetic Resonance Imaging Distortion Correction

**Figure 1:**
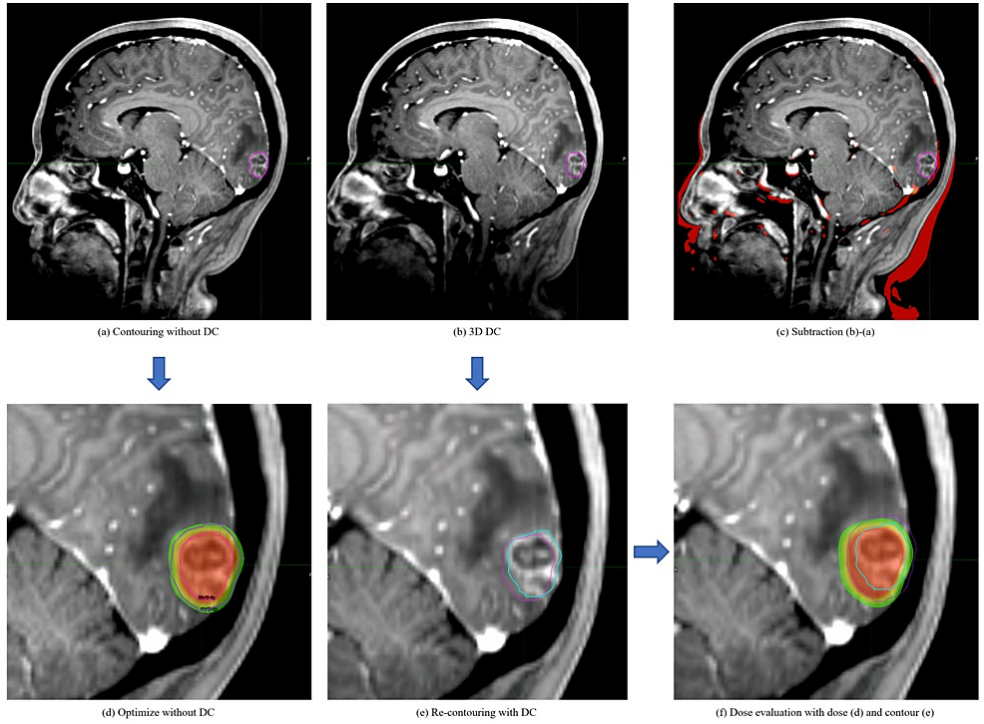
Dose distribution comparison between tumor delineation with and without distortion correction (DC) (a) GTV on MRI without DC; (b) How GTV contoured on MRI without DC (pink line) looks like on MRI with 3D-DC. GTV is not covering some parts, especially the outer part, of the contrast-enhanced tumor; (c) Geometric difference between (a) and (b). The gratitude of the distortion depends on the distance from the central axis; (d) A stereotactic radiotherapy plan optimized based on MRI without DC showing beautiful coverage of the GTV and PTV; (e) Re-contouring performed on MRI with DC (blue). 2-3 mm difference is captured; (f) Actual dose distribution the patient will receive if the plan was made on MRI without DC. PTV coverage and dose to GTV are both inadequate.

**Figure 2:**
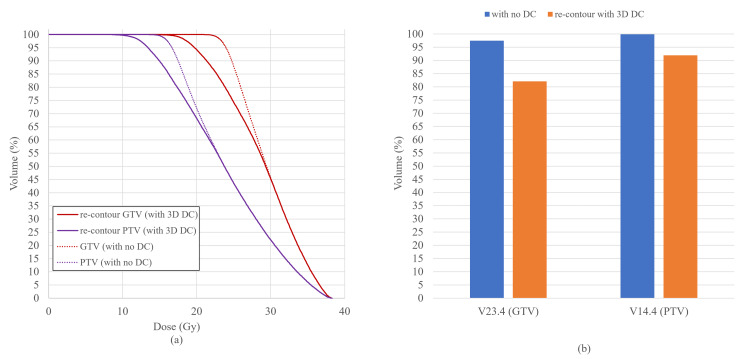
Dose-volume histogram (DVH) and dose coverage of PTV and GTV (a) Dotted line showing the DVH of PTV and GTV of plan optimized based on MRI without DC (Figure 1d) and solid line showing DVH of PTV and GTV the patient will receive (Figure 1f). (b) V23.4Gy of GTV and V14.4Gy of PTV are shown. If the treatment plan was optimized on MRI without DC (blue), actual GTV and PTV (re-contoured on MRI with DC) will not be covered as planned (orange).

Treatment plans using MRI with and without DC were compared using MIM maestro ver. 7.2.3 (MIM Software Inc., Beachwood, Ohio). The MR images without DC were fused to the planning CT, and the SRT plan was created based on the PTV created with MRI without DC. The dose distribution of the plan without DC with reference to PTV with DC is shown in Figure 1. Figure 2 shows how the dose-volume histogram will be affected.

Outcome of the Patient

The patient completed the scheduled treatment without any acute side effects. The irradiated tumor responded to treatment; however, after four months, the patient had developed new brain metastases with bleeding and has since died of the disease.

## Discussion

Distortions on MRI are sometimes difficult to identify. During treatment planning, failure to recognize the presence of spatial distortions in the image can lead to the mistaken belief that the correct tumor contouring has been achieved. At our hospital, CyberKnife is often used for SRT planning of brain metastases, which allows irradiation within 1 mm of the spatial recognition of the planned CT and allows a higher dose to the tumor while minimizing the dose to the surrounding normal organs. However, if distortion causes a deviation of more than 1 mm, a higher dose may be delivered to the normal tissue and an insufficient dose to the tumor. Although there are many reports of distortion in basic validation [7-9], nothing is more effective than visually alerting clinicians to how much misidentification can occur in real-world clinical practice. Therefore, we report this case to show how difficult it is to recognize distortion and the degree of treatment error it can cause. We have previously studied and reported in detail the misalignment associated with distortion between the planning CT and the MRI employed at our clinic. We have found that when treatment planning is performed using MRI without 3D DC, distortion becomes non-negligible at a distance of about 100 mm from the central axis. 2D DC can improve spatial accuracy in the in-plane direction but not enough in the through-plane direction. The 3D DC is indispensable for correcting 3D volume spatial distortion, and this is extremely important if we are using MRI for treatment planning because we are prescribing dose in volume. Without DC, a misalignment of about 5 mm can be seen at a distance of 150 mm from the central axis. This misalignment will be suppressed to about 3-4mm if vendor-specific 2D DC was used, however, if 3D DC was used, it was suppressed to about 1 mm [11].

Some may argue that we should add some PTV margin for intra and inter-fraction errors and that misalignment caused by distortion may be included within this margin. However, a 5-mm margin for brain tumors is too large. Further, in many countries with limited medical resources, it is not easy to repeat MRI for treatment planning; therefore, using diagnostic MRI for treatment planning is understandable. If the axial center is close to the tumor, distortion on MRI can be minimized. However, when diagnostic MRI is used for treatment planning, the location of metastases is unknown at the time of examination, and it is impractical to adjust the axis center each time. Although 2D DC is often used in the field of diagnosis, the importance of 3D distortion correction is not well recognized. However, we should be fully aware of 3D distortion and understand that 3D DC allows for high-precision treatment using diagnostic MRI.

## Conclusions

In this case report, we have visually addressed the importance of 3D DC on MRI when using MRI for SRT planning. Distortion on MRI can be difficult to recognize, but we should always be aware of it. When we are planning high-precision radiotherapy with MRI references, we should always use 3D DC to assure that we are treating in the correct location.

## References

[1] (2022). National Comprehensive Cancer Network, Central Nervous System Cancer (Version 1.2022). https://www.nccn.org/professionals/physician_gls/pdf/cns.pdf.

[2] Patil CG, Pricola K, Sarmiento JM, Garg SK, Bryant A, Black KL (2017). Whole brain radiation therapy (WBRT) alone versus WBRT and radiosurgery for the treatment of brain metastases. Cochrane Database Syst Rev.

[3] Yamamoto M, Serizawa T, Sato Y, Higuchi Y, Kasuya H (2021). Stereotactic radiosurgery results for patients with 5-10 versus 11-20 brain metastases: a retrospective cohort study combining 2 databases totaling 2319 patients. World Neurosurg.

[4] Tamari K, Suzuki O, Hashimoto N (2015). Treatment outcomes using CyberKnife for brain metastases from lung cancer. J Radiat Res.

[5] Xuyao Y, Zhiyong Y, Yuwen W (2020). Improving stereotactic radiotherapy (SRT) planning process for brain metastases by Cyberknife system: reducing dose distribution in healthy tissues. J Cancer.

[6] Prabhakar R, Haresh KP, Ganesh T, Joshi RC, Julka PK, Rath GK (2007). Comparison of computed tomography and magnetic resonance based target volume in brain tumors. J Cancer Res Ther.

[7] Pappas EP, Alshanqity M, Moutsatsos A (2017). MRI-related geometric distortions in stereotactic radiotherapy treatment planning: evaluation and dosimetric impact. Technol Cancer Res Treat.

[8] Putz F, Mengling V, Perrin R (2020). Magnetic resonance imaging for brain stereotactic radiotherapy: a review of requirements and pitfalls. Strahlenther Onkol.

[9] Nakazawa H, Mori Y, Komori M, Shibamoto Y, Tsugawa T, Kobayashi T, Hashizume C (2014). Validation of accuracy in image co-registration with computed tomography and magnetic resonance imaging in Gamma Knife radiosurgery. J Radiat Res.

[10] Theocharis S, Pappas EP, Seimenis I, Kouris P, Dellios D, Kollias G, Karaiskos P (2022). Geometric distortion assessment in 3T MR images used for treatment planning in cranial stereotactic radiosurgery and radiotherapy. PLoS One.

[11] Kato Y, Okudaira K, Kamomae T (2022). Evaluation of system-related magnetic resonance imaging geometric distortion in radiation therapy treatment planning: two approaches and effectiveness of three-dimensional distortion correction [Article in Japanese]. Nagoya J Med Sci.

